# Ischemia–Reperfusion Injury and Immunosuppressants Promote Polyomavirus Replication Through Common Molecular Mechanisms

**DOI:** 10.3389/fimmu.2022.835584

**Published:** 2022-02-25

**Authors:** Xu-Tao Chen, Yang Huang, Jing Wang, Ge Li, Yu Zhang, Li-Fang He, Yue-Xiao Lian, Shi-Cong Yang, Guo-Dong Zhao, Hui Zhang, Jiang Qiu, Lei Zhang, Gang Huang

**Affiliations:** ^1^ Department of Organ Transplant, The First Affiliated Hospital of Sun Yat-sen University, Guangzhou, China; ^2^ Guangdong Provincial Key Laboratory of Laboratory Animals, Guangdong Laboratory Animals Monitoring Institute, Guangzhou, China; ^3^ Department of Pathology, The First Affiliated Hospital of Sun Yat-sen University, Guangzhou, China; ^4^ Department of Organ Transplant, The Second Affiliated Hospital of Guangzhou Medical University, Guangzhou, China

**Keywords:** mouse polyomavirus, immunosuppressants, renal ischemia–reperfusion injury, WGCNA, pathway enrichment analysis, NF-κB

## Abstract

**Background:**

BK polyomavirus (BKPyV)-associated nephropathy (BKPyVAN) causes renal allograft dysfunction and graft loss. However, the mechanism of BKPyV replication after kidney transplantation is unclear. Clinical studies have demonstrated that immunosuppressants and renal ischemia–reperfusion injury (IRI) are risk factors for BKPyV infection. Studying the pathogenic mechanism of BKPyV is limited by the inability of BKPyV to infect the animal. Mouse polyomavirus (MPyV) is a close homolog of BKPyV. We used a model of MPyV infection to investigate the core genes and underlying mechanism of IRI and immunosuppressants to promote polyomavirus replication.

**Materials and Methods:**

One-day-old male C57BL/6 mice were intraperitoneally injected with MPyV. At week 9 post-infection, all mice were randomly divided into IRI, immunosuppressant, and control groups and treated accordingly. IRI was established by clamping the left renal pedicle. Subsequently, kidney specimens were collected for detecting MPyV DNA, histopathological observation, and high-throughput RNA sequencing. Weighted gene correlation network analysis (WGCNA), protein–protein interaction network analysis, and Kyoto Encyclopedia of Genes and Genomes (KEGG) pathway enrichment analysis were used to screen for core genes and common signaling pathways involved in promoting MPyV replication by IRI and immunosuppressants.

**Results:**

After primary infection, MPyV established persistent infection in kidneys and subsequently was significantly increased by IRI or immunosuppressant treatment individually. In the IRI group, viral loads peaked on day 3 in the left kidney, which were significantly higher than those in the right kidney and the control group. In the immunosuppressant group, viral loads in the left kidney were significantly increased on day 3, which were significantly higher than those in the control group. Protein–protein interaction network analysis and WGCNA screened complement C3, epidermal growth factor receptor (EGFR), and FN1 as core genes. Pathway enrichment analysis based on the IRI- or immunosuppressant-related genes selected by WGCNA indicated that the NF-κB signaling pathway was the main pathway involved in promoting MPyV replication. The core genes were further confirmed using published datasets GSE47199 and GSE75693 in human polyomavirus-associated nephropathy.

**Conclusions:**

Our study demonstrated that IRI and immunosuppressants promote polyomavirus replication through common molecular mechanisms. In future studies, knockdown or specific inhibition of C3, EGFR, FN1, and NF-κB signaling pathway will further validate their critical roles in promoting polyomavirus replication.

## Introduction

BK polyomavirus (BKPyV) is a member of the polyomavirus family, with a small circular double-stranded DNA genome of 5,300 base pairs. The polyomavirus family includes the homologous JC polyomavirus in humans, simian virus 40 in non-human primates, and mouse polyomavirus (MPyV) in mice. BKPyV is mainly activated in immunocompromised individuals and causes BKPyV-associated nephropathy (BKPyVAN), especially in kidney transplant recipients. BKPyVAN is an important cause of renal allograft dysfunction and graft loss. However, our understanding of the pathogenesis of BKPyVAN and the development of effective therapies for BKPyVAN has been limited. The mechanism of BKPyV reactivation and replication after kidney transplantation is still unclear.

BKPyV infection in kidney transplant recipients may be caused by a combination of multiple factors simultaneously. Uncontrollable clinical factors make it difficult to directly study the pathogenesis of BKPyV in humans. To better explore the factors that promote BKPyV replication and the molecular mechanisms involved, studies can only be conducted with the help of animal models. However, the Polyomaviridae family has strong species specificity. BKPyV exhibits a very narrow host range. The inability of BKPyV to cause productive infections in animals other than humans results in the impossibility to directly investigate the pathogenesis of BKPyV reactivation and BKPyVAN in animal models.

MPyV is a close homolog of BKPyV. Both of them have a small circular double-stranded DNA genome of about 5,300 base pairs, and they are more than 70% genetically homologous (1). They both have structurally similar non-coding regions and early and late coding regions and are highly similar in viral gene transcription and viral protein synthesis. MPyV mimics human polyomavirus in many ways. They enter a long-life latent phase after infecting the host during infancy and reactivate under the immunosuppression status. Furthermore, the pattern of distribution of the infected organs and tissues is similar in the two species (2). They mainly infect the urinary epithelium. There is great commonality between BKPyV and MPyV in terms of the mechanism by which the viruses enter host cells. They both bind to the GT1b receptor on the cell surface and enter the cell in an endocytosis-dependent manner and then replicate in the nucleus and release progeny virus particles. These biological similarities suggest that the mouse model of MPyV infection can be used to study the pathogenesis of human BKPyV infection. Han lee et al. established a mouse model of MPyV infection to mimic the BKPyVAN that occurred in human kidney transplant recipients. Their results showed that acute MPyV infection in renal allograft augmented the alloreactive CD8+ T-cell response while maintaining the antiviral CD8+ T-cell response (3). Using a mouse model of MPyV infection, Albrecht et al. demonstrated that the polyomavirus-associated allograft injury was mainly caused by the interplay between viral infection and anti-donor immune response (2). Similarly, Steven et al. found that alloimmune T-cell response induced by MPyV-caused inflammation/injury was the major culprit of polyomavirus-associated allograft injury in mice (2). Therefore, in this study, we established a mouse model of MPyV infection by intraperitoneal injection to simulate BKPyV infection in humans.

Large prospective clinical studies have highlighted that immunosuppressants (IS), especially tacrolimus and mycophenolic acid, and renal ischemia–reperfusion injury (IRI) are risk factors for BKPyV infection after kidney transplantation. In clinical scenarios, kidney transplant recipients often suffer from both IRI and IS simultaneously, making it impossible to separately evaluate the effects of IRI and IS on promoting polyomavirus replication in humans. In the present study, we separately administered IRI and IS treatment to a mouse model of MPyV infection to assess the role of each factor in promoting polyomavirus replication. High-throughput RNA sequencing (RNA-seq) and weighted gene correlation network analysis (WGCNA) were used to screen for core genes involved in the process of promoting MPyV replication by IRI and IS. Finally, common signaling pathways of IRI and IS promoting MPyV replication were obtained by Kyoto Encyclopedia of Genes and Genomes (KEGG) pathway enrichment analysis.

## Materials and Methods

### Experimental Mice

Newborn male C57BL/6 mice were provided by Guangdong Laboratory Animals Monitoring Institute and housed in specific pathogen-free animal facilities. All experiments involving animals were conducted following the National Guidelines for Ethics Review of Laboratory Animal Welfare. The study was approved by the Ethics Committee of Guangdong Laboratory Animals Monitoring Institute (No. I-IACUC2019006).

### Virus Culture, Isolation, and Measurement

#### Virus Culture

The culture method refers to our previous published paper (4). MPyV LID-1 strain (ATCC VR-252) was propagated in BHK-ATCC cells. After 1-h adsorption, a serum-free Dulbecco’s modified Eagle medium (DMEM) was added. The cultures were then incubated in a 37°C/5% CO_2_ humidified incubator. When 70% of the cells showed obvious cytopathic effects, the cultures were harvested, thawed, and refrozen three times. Subsequently, samples were centrifuged for 10 min at 4,000 rpm, and the supernatant was collected.

#### Virus Isolation and Purification

The supernatant was transferred to an ultracentrifuge tube and spun at 41,000 rpm for 2.5 h at 4°C. After that, the cell-free supernatant was discarded, and the bottom sediment was re-suspended and washed with sterile phosphate-buffered saline (PBS). Sucrose solutions with gradient concentrations of 10%, 25%, 45%, and 60% were prepared with PBS. Three milliliters of 10%, 3 ml of 25%, 2 ml of 45%, and 2 ml of 60% sucrose solution were added to the centrifuge tubes. Subsequently, the virus solution was then transferred on top of the sucrose solution. Samples were centrifuged at 27,000 rpm for 3 h at 4 °C in an SW41 rotor. After centrifugation, three distinct white bands from the interfaces at 10%–25%, 25%–45%, and 45%–60% sucrose layers were aspirated and subsequently diluted with PBS. Samples were centrifuged at 27,000 rpm for 2 h in an SW41 rotor. After centrifugation, the supernatant was discarded. The precipitate was re-suspended with PBS and then stored in a refrigerator at −80°C.

#### Detection and Quantification of Viral Load

Viral load was detected by fluorescence quantitative PCR. Under the established quality control condition, samples were considered negative for MPyV DNA if there was no fluorescence amplification curve but defined as positive if fluorescence amplification curve was observed and Ct value ≤35.

### Mouse Polyomavirus Infection Mouse Model

In this study, there were two independent batches of mice. The first batch of mice was mainly used to verify whether MPyV could establish latent infection in mice. One-day-old male C57BL/6 mice were intraperitoneally injected with 30 μl of MPyV (10^5^ copies/μl). On 0, 3, 10, 24, 35, 60, 80, and 100 days post-injection, three mice were randomly sacrificed at each time point, and blood and kidney specimens were collected for detecting MPyV DNA.

**Figure 1:**
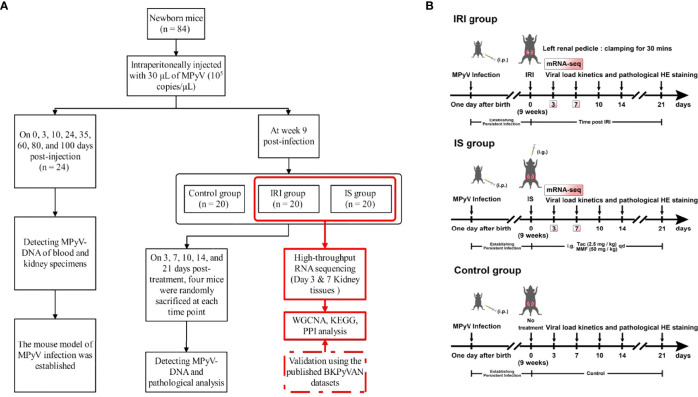
**(A)** The flowchart of experimental design. **(B)** Schematic of experimental setup. One-day-old male C57BL/6 mice were intraperitoneally injected with 30 μl of MPyV (10^5^ copies/μl). All mice were individually housed in a biosafety level 2 room until week 9. IRI was established by clamping the left renal pedicle for 30 min. IS treatment was achieved by gastric gavage of tacrolimus (2.5 mg/kg) and mycophenolic acid (50 mg/kg) daily. The control group did not receive any treatment. MPyV, mouse polyomavirus; IRI, ischemia–reperfusion injury; IS, immunosuppressant; BKPyVAN, BK polyomavirus-associated nephropathy; WGCNA, weighted gene correlation network analysis; KEGG, Kyoto Encyclopedia of Genes and Genomes; PPI, protein–protein interaction; mRNA-seq, messenger RNA sequencing.

Subsequently, we established intrarenal MPyV latent infection in the second batch of newborn mice using the method described above. At week 9 post-infection, all mice were randomly divided into IRI (n = 20), IS (n = 20), and control (n = 20) groups and treated accordingly. In the IRI group, IRI was established by clamping the left renal pedicle for 30 min. In the IS group, tacrolimus (2.5 mg/kg) and mycophenolic acid (50 mg/kg) were administered by gastric gavage once daily. No intervention was done in the control group. The animal model and experimental design are shown in **Figure 1**. On 3, 7, 10, 14, and 21 days post-treatment, four mice were randomly sacrificed at each time point according to the predesigned labeling method, and kidney specimens were collected. Plasma and kidney specimens for MPyV-DNA testing were stored at −20°C. Kidney specimens for high-throughput RNA-seq were stored in liquid nitrogen. In this study, direct evidence of MPyV replication in kidney tissue was detected by quantitative PCR for MPyV DNA.

### Histological Evaluation

Kidneys were fixed in 10% neutral-buffered formalin and embedded in paraffin. The paraffin sections of 3-μm thickness were stained with H&E. The slides were observed by an experienced renal pathologist (S-CY) in a single-blind fashion to evaluate the morphology evidence of IRI and polyomavirus infection. IRI mainly presented as acute renal tubular injury, which was defined as tubular dilatation, epithelial swelling, flattening, sloughing, or coagulative necrosis in non-atrophic tubules. MPyV infection was histopathologically defined as the presence of viral inclusion bodies.

### RNA Isolation, Library Preparation, and Sequencing

Deep sequencing of transcriptional profiling in kidney tissues was performed on day 3 and day 7 in the IRI and IS groups, respectively. For RNA-seq analysis, each RNA sample was run in triplicate. Total RNAs were extracted from samples using the RNeasyMini Kit (Qiagen, Hilden, Germany). RNA quality was assessed by using NanoDrop (Thermo Fisher Scientific, Waltham, MA, USA) and Qubit Fluorometer (Invitrogen, Carlsbad, CA, USA). High-quality RNA samples (28 S/18 S = 2.0–2.2, RIN > 9.0) were used to construct sequencing libraries following a standardized paired-end read procedure on the Illumina HiSeq 2000 Sequencing platform (KeyGEN, Beijing, China). The raw data in FASTQ format were processed using fastp 0.20.0 (5). Clean data (clean reads) were obtained after quality control, adapter trimming, quality filtering, and per-read quality cutting. All downstream analyses were performed on high-quality clean data. Gene expression was quantified using Salmon software 0.11.2 (6). The high-throughput RNA sequencing data reported in this study have been deposited in the Gene Expression Omnibus database (GSE192576).

### Weighted Gene Correlation Network Analysis

WGCNA was performed to select the gene modules significantly associated with IRI, IS treatment, and virus. A total of 8,550 genes in the top 35% of variance were screened from the data set to build gene networks using a scale-free topology model, using the R package WGCNA (7). In brief, we selected the power β = 6 for the weighting correlation matrix following an approximate scale-free topology criterion. Subsequently, gene expression modules with similar expression patterns were identified based on the gene cluster dendrogram and by using the dynamic tree cut method (minModuleSize = 50, mergeCutHeight = 0.3, deepSplit = 1). The unsigned network was used to build the relationships between modules and phenotypes. To identify modules that were significantly associated with the traits of samples, the module eigengenes were calculated and correlated with the groups. Modules with a *p* < 0.05 and Pearson’s correlation r > 0.4 were considered significantly correlated to the sample trait. Genes in the significantly correlated modules were considered to be putative genes related to the group.

### Pathway Enrichment Analysis

KEGG is a database for systematic analysis of gene function, which links genomic information with higher-level function information (8). After genes correlated to the phenotypes by WGCNA were obtained, the R package clusterProfiler (9) was used to perform KEGG pathway over-representation analysis, at a cutoff of *p* < 0.05.

### Protein–Protein Interaction Analysis

The mouse protein–protein interaction (PPI) network used in this analysis was retrieved from the STRING database (version 11.0), with medium confidence >0.7. The PPI network was constructed with genes in the Modules significantly correlated with clinical trials and was visualized using Cytoscape software (version 3.7.1). Hub genes of PPI networks are highly connected nodes with particular biological properties and are more conserved than non-hubs. Hub genes were calculated and identified by Closeness algorithms using cytoHubba analysis (10). Finally, the top 10 genes conditioned on MNC, EPC, and Closeness were screened separately.

### Validation Using the Published BK Polyomavirus-Associated Nephropathy Datasets

To test whether the core genes screened in mice with MPyV infection are also expressed in human BKPyVAN, we downloaded two microarray datasets from the Gene Expression Omnibus database at the National Center for Biotechnology Information (https://www.ncbi.nlm.nih.gov/geo/) and identified differentially expressed genes by using the limma package. The microarray dataset GSE75693 included 30 patients with stable kidney transplantation and 15 with BKPyVAN (11). The dataset GSE47199 contained three BKPyVAN biopsies and 11 normal biopsies (12).

### Statistical Analyses

Continuous data were presented as mean ± SD if normally distributed, or as median and interquartile range if non-normally distributed. Categorical variables were presented as frequency (percentage). Two groups were compared using Student’s t-test and multiple groups with the one-way ANOVA and least significant difference (LSD) tests if normality was assumed. If non-normality was assumed, the Mann–Whitney U test (two independent groups) or the Kruskal–Wallis test (multiple groups) was used. For comparing pre- versus post-treatment, paired t-test was used. Repeated measurements were compared by repeated-measures ANOVA. All statistical analyses were 2-tailed, and *p*-value <0.05 was considered statistically significant. Statistical analyses were performed using SPSS version 20 (IBM, Armonk, NY, USA) and GraphPad Prism 8 (GraphPad, La Jolla, CA, USA).

## Results

### Persistent Mouse Polyomavirus Infection in Kidney of C57BL/6 Mice

**Figure 2:**
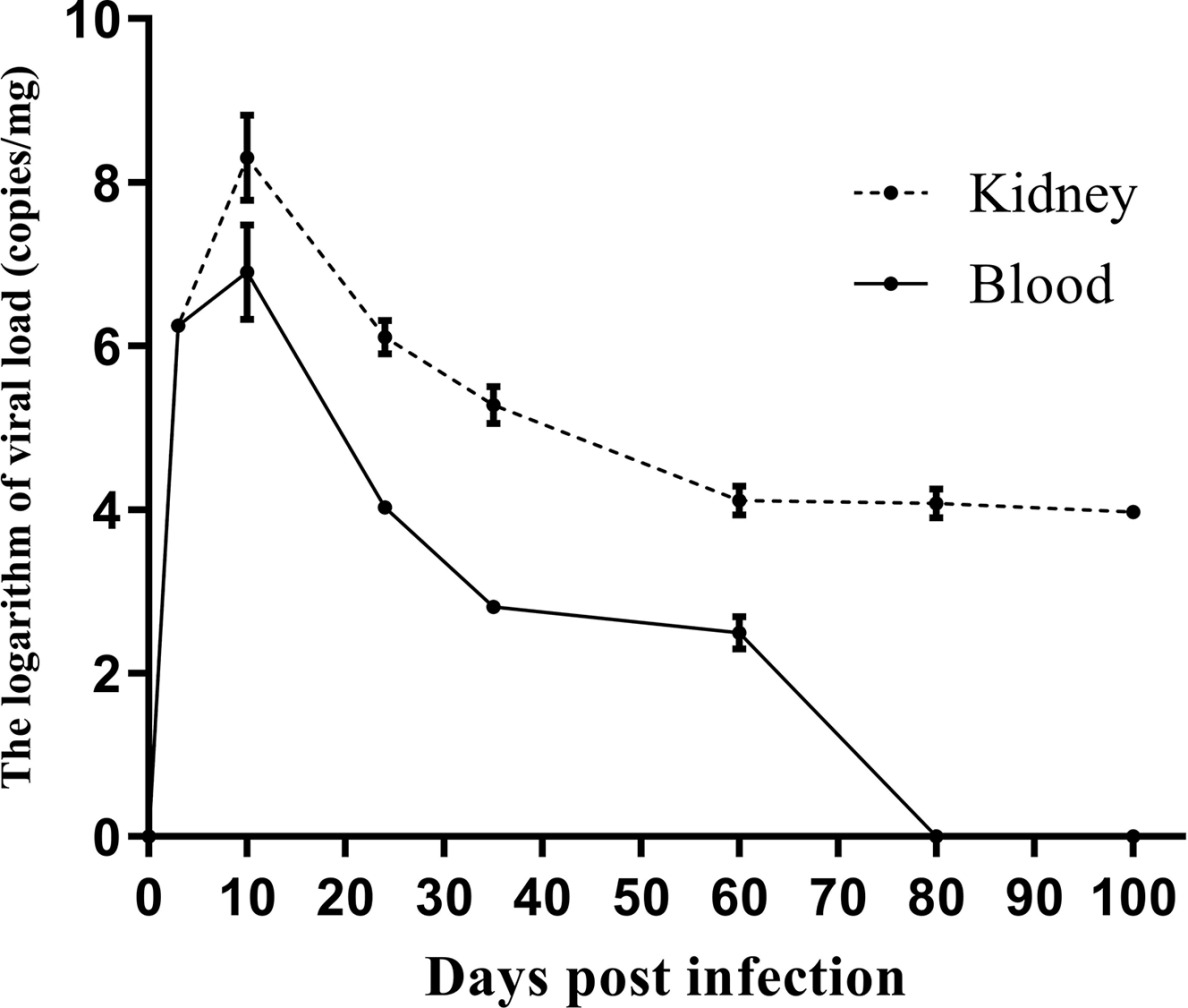
Kinetics of left kidney and plasma viral loads in C57BL/6 mice at multiple time points after primary mouse polyomavirus infection. Error line indicates mean and SD.

**Figure 2** shows the viral dynamics in the left kidney and blood after primary infection. MPyV viral loads in the left kidney and blood peaked on day 12 and gradually decreased. MPyV viral loads in the kidney were maintained at approximately 10^4^ copies/mg at and beyond week 9, while MPyV viral loads in the blood dropped below the detection threshold after 80 days. **Figure 2** demonstrates that MPyV latent infection was successfully established in mice.

### Ischemia–Reperfusion Injury Promoted Mouse Polyomavirus Replication in the Kidney

**Figure 3:**
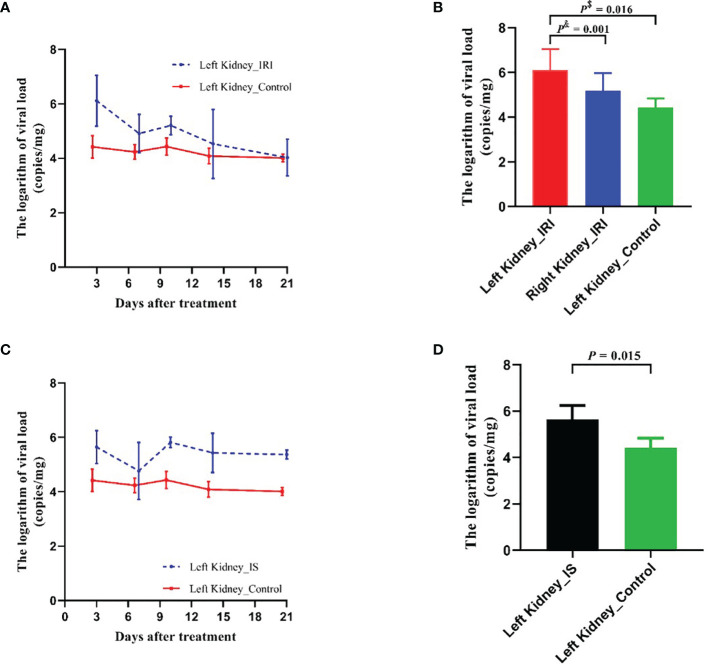
Comparison of MPyV viral loads in kidneys between IRI group, IS group, and control group. **(A)** Dynamic comparison of viral loads in left kidneys between IRI group and control group. Error line indicates mean and SD. *p* ^$^ was compared by Student’s t-test. *p* ^&^ was compared by paired t-test. **(B)** Comparison of viral loads in left kidney and right kidney of IRI group, as well as in left kidney of control group on day 3 after IRI treatment. **(C)** Dynamic comparison of viral loads in left kidneys between IS (tacrolimus + mycophenolic acid) group and control group. Error line indicates mean and SD. **(D)** Comparison of viral loads in left kidneys of IRI group, IS group, and control group on day 3 after treatment. MPyV, mouse polyomavirus; IRI, ischemia–reperfusion injury; IS, immunosuppressant.

**Figure 4:**
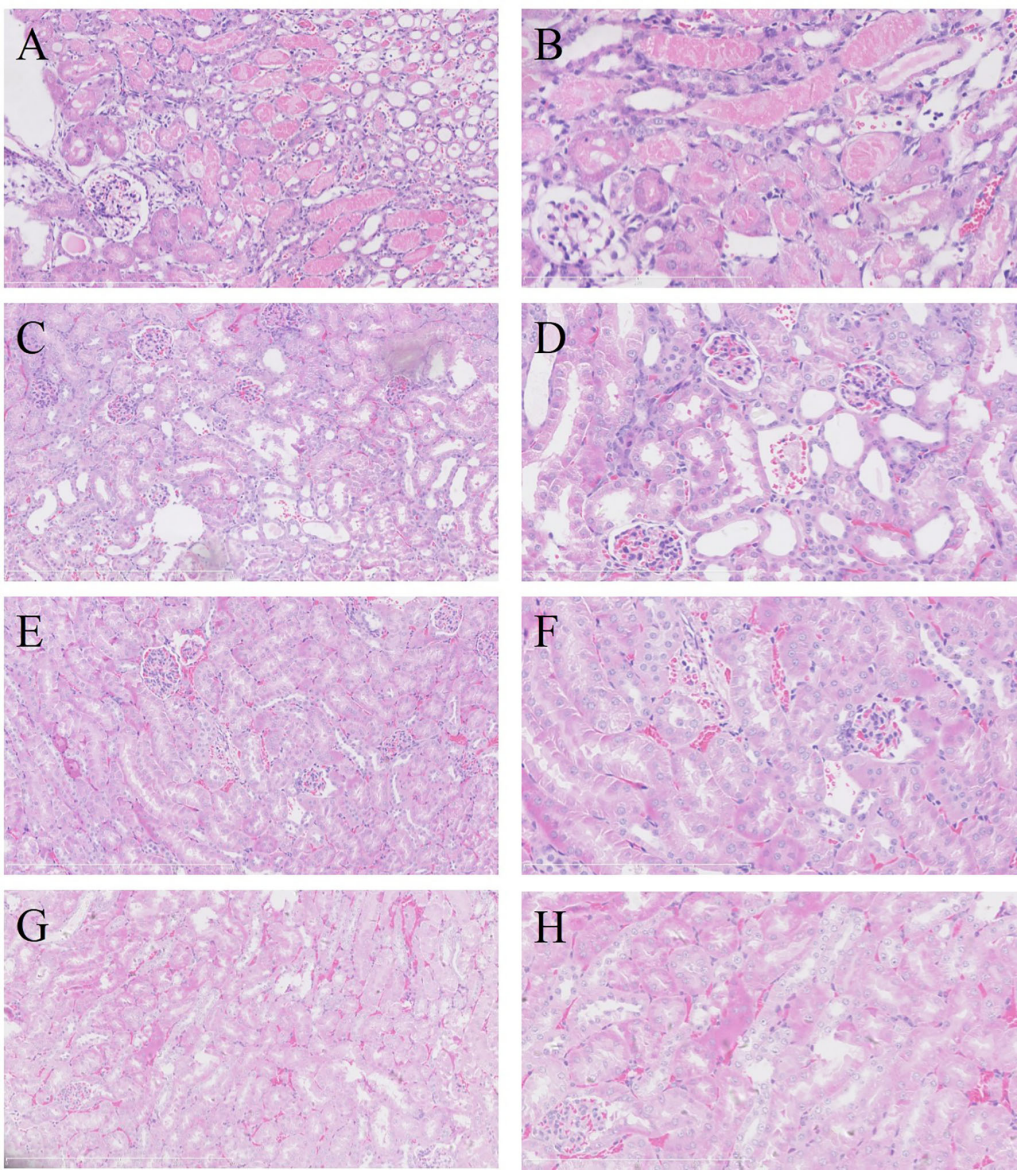
Renal pathological manifestations in each group on day 3 after treatment (H&E staining). In the left kidney of IRI group, severe acute tubular injury showed swelling of the proximal tubular epithelium (**A**, ×200; **B**, ×400). Coagulative necrosis was identified in foci of tubular epithelium, especially in the juxtamedullary area. No acute tubular injury was observed in the right kidney of IRI group (**C**, ×200; **D**, ×400) or in the left kidney in IS group (**E**, ×200; **F**, ×400) and control group (**G**, ×200; **H**, ×400). IRI, ischemia–reperfusion injury; IS, immunosuppressant.

For evaluating the effect of IRI on MPyV replication, 9-week-old mice were subjected to IRI by clamping left renal hilar for 30 min. MPyV viral loads in the left kidney significantly increased after IRI, peaked on day 3, and gradually decreased (**Supplementary Table 1**). In the control group, viral loads in the left kidney were maintained at approximately 10^4^ copies/mg, where no time trend was observed (repeated-measures ANOVA, *p* = 0.196) (**Figure 3A**). On day 3 after IRI, viral loads in the left kidney of the IRI group were significantly higher than those in the right kidney (6.118 ± 0.467 vs. 5.180 ± 0.395 log_10_ copies/mg, *p* = 0.001) and those in the left kidney of the control group (6.118 ± 0.467 vs. 4.420 ± 0.207 log_10_ copies/mg, *p* = 0.016), respectively. Viral loads of the right kidney in the IRI group were comparable to those in the left kidney of the control group (5.180 ± 0.395 vs. 4.420 ± 0.207 log_10_ copies/mg, *p* = 0.139) (**Figure 3B**). We dynamically evaluated renal pathological changes after IRI (**Supplementary Figure 1**) and found that the process of kidney damage/repair is consistent with the dynamics of intrarenal MPyV viral loads. On day 3 after IRI, typical tubular injuries were observed in the left kidney but not in the right kidney. No obvious pathological injury was identified in the IS group or control group on day 3 (**Figure 4**).

### Immunosuppressants Promoted Mouse Polyomavirus Replication in the Kidney

For evaluating the effect of IS on MPyV replication, tacrolimus and mycophenolic acid were intragastrically administrated to the 9-week-old mice daily. After treatment, MPyV viral loads in the kidneys significantly increased and maintained approximately 10^5^–10^6^ copies/mg (**Supplementary Table 1**). Viral loads in the left kidney of the IS group were significantly higher than those of control group at days 3 (*p* = 0.015), 10 (*p* < 0.001), 14 (*p* = 0.014), and 21 (*p* < 0.001) after treatment, but no difference was observed at day 7 (*p* = 0.360) (**Figure 3C**). On day 3 after IS treatment, viral loads in the left kidney of the IS group were higher than those of the control group (5.643 ± 0.602 vs. 4.420 ± 0.414 log_10_ copies/mg, *p* = 0.015) (**Figure 3D**).

### Weighted Gene Co-Expression Network Construction and Analysis

**Figure 5:**
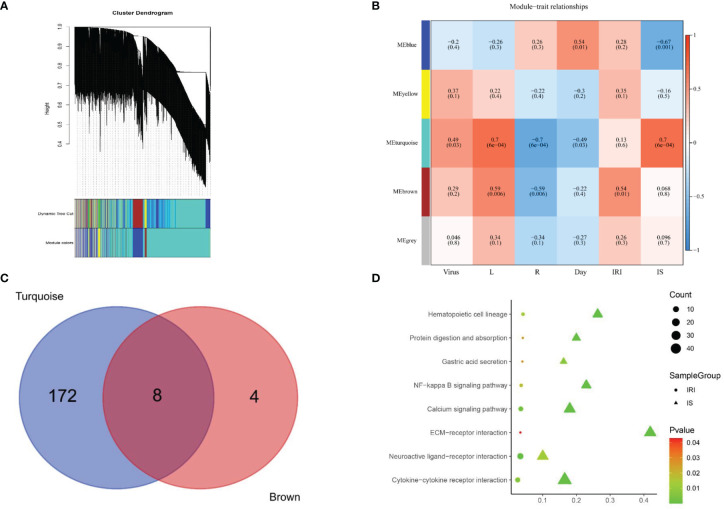
Signaling pathways screening. **(A)** Gene dendrogram was generated using average linkage hierarchical clustering. The dynamic tree cut yielded five modules. Module colors were displayed correspondingly. **(B)** Heatmap of module–trait relationships depicting correlations between module genes and variables. Numbers in the table correspond to the correlation r, and the *p*-value is in parentheses. The degree of correlation is illustrated with the color legend. **(C)** Venn diagram showing the overlapping genes of Meturquoise module and Mebrown modules. Eight core genes are shared between 180 expressed genes of mouse polyomavirus infection and IS treatment and 12 expressed genes of IRI treatment. **(D)** KEGG pathway analysis of the genes in the Meturquoise module and the Mebrown module. L, left kidney; R, right kidney; IRI, ischemia–reperfusion injury; IS, immunosuppressant; KEGG, Kyoto Encyclopedia of Genes and Genomes.

In this study, the power of β = 6 was selected as the soft threshold according to the scale-free topology criterion. The topological overlap measure (TOM) was calculated using the adjacency matrix. Based on TOM, a hierarchical clustering tree (dendrogram) of genes was produced using the hierarchical clustering function. We clustered 8,550 genes into five distinct modules using hierarchical clustering followed by dynamic tree cutting (**Figure 5A**). The Mebrown module (479 genes) was significantly associated with IRI (r = 0.54, *p* = 0.01), while the Meturquoise module (2,784 genes) was associated with IS as well as viral replication (r = 0.7, *p* = 0.0001) (**Figure 5B**). Genes that cannot be included in any module were added to the gray module and rejected in subsequent analyses.

### Kyoto Encyclopedia of Genes and Genomes Pathway Analysis

KEGG pathway analysis revealed that 180 pathways were enriched in the Meturquoise module and 12 pathways in the Mebrown module. The enriched pathways in both modules were mapped to each other to generate eight common pathways associated with IRI, IS, and viral replication (**Figures 5C, D**), including 1) gastric acid secretion, 2) NF-kappa B signaling pathway, 3) cytokine–cytokine receptor interaction, 4) neuroactive ligand–receptor interaction, 5) extracellular matrix (ECM)–receptor interaction, 6) protein digestion and absorption, 7) hematopoietic cell lineage, and 8) calcium signaling pathway.

### Protein–Protein Interaction Network Construction and Core Gene Screening

**Figure 6:**
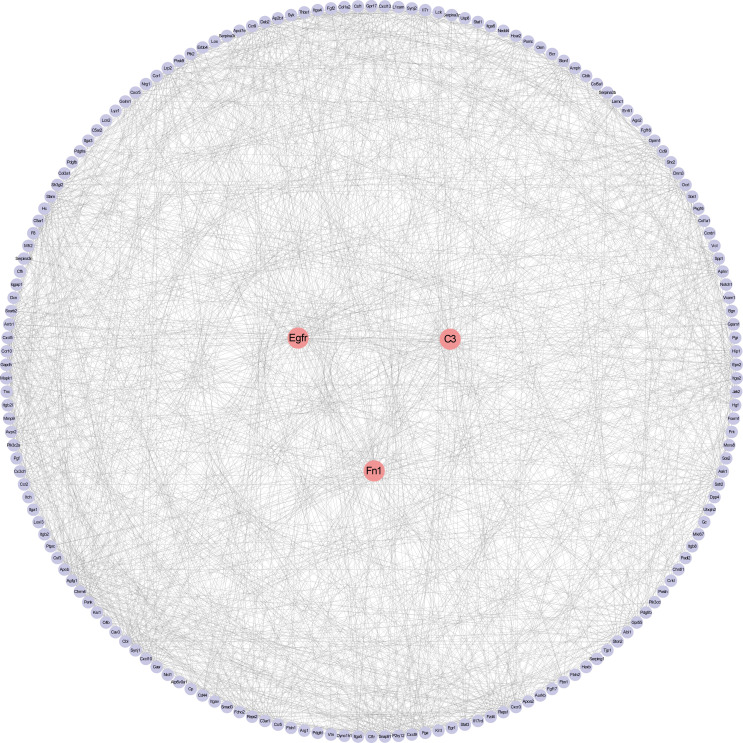
Protein–protein interaction network construction and analysis. Red nodes represent core genes. Others were the first nodes of core genes.

The PPI network upon the genes from the Meturquoise and Mebrown modules was presented in **Figure 6**. The top 10 genes were further screened by combining the three local-based methods (MNC, EPC, and Closeness) in the Cytoscape plug-in cytoHubba. Three core candidate genes were obtained by taking the intersection of hub genes in the PPI network, including complement C3, epidermal growth factor receptor (EGFR), and fibronectin 1 (FN1). The relationship between the hub genes and the pathways is shown in **Supplementary Table 2**.

### Validation of Core Genes and Signaling Pathways

**Figure 7:**
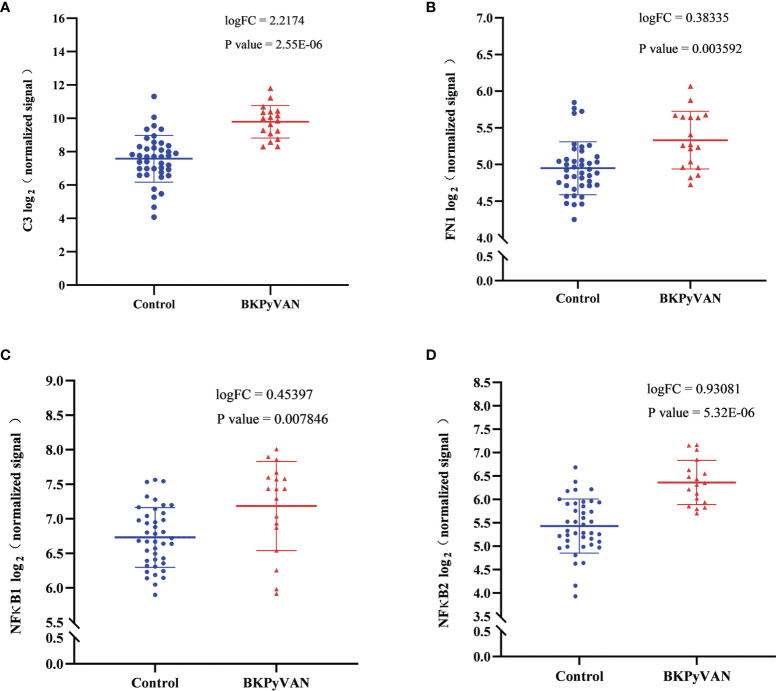
Microarray datasets (GSE47199 and GSE75691) from BKPyVAN and normal transplanted kidneys were integrated into descending batches. The core genes, C3 **(A)**, FN1 **(B)**, NF-κB1 **(C)**, and NF-κB2 **(D)** identified in this study, were compared between BKPyVAN and normal transplanted kidney using R packages limma. BKPyVAN, BK polyomavirus-associated nephropathy; C3, complement 3; NF-κB, NF-kappa B; FN1, fibronectin 1.

We verified the expression of the above core genes in BKPyVAN from published databases. The results showed that C3, FN1, NF-κB1, and NF-κB2 were significantly more highly expressed in BKPyVAN compared with normal transplanted kidneys (**Figure 7**). EGFR was also higher in BKPyVAN than in controls, although a statistically significant difference was not reached (5.101 ± 0.371 vs. 4.898 ± 0.370, *p* = 0.112).

## Discussion

Numerous clinical studies have described multiple clinical factors associated with BKPyV infection after kidney transplantation (13, 14). Of these, IRI and IS were proved to be critical risk factors. However, it is difficult to individually verify the effect of IRI and IS because they occur in every kidney transplant recipient. To eliminate the potential confounding biases, we successfully established a mouse model of MPyV infection and separately demonstrated that either renal IRI or IS was sufficient to promote MPyV replication in kidneys. Furthermore, we found that IRI and IS may stimulate MPyV replication through common molecular mechanisms, mainly including EGFR, C3, FN1, and NF-κB signaling pathways. More importantly, microarray datasets of BKPyVAN from two studies (11, 12) also supported our findings, which fully illustrated the extrapolation of our results.

Seroepidemiologic studies show that BKPyV establishes lifelong latent infection in more than 80% of hosts after primary infection (15). In this respect, our model of preliminarily established persistent MPyV infection followed by reactivation because IRI or IS was close to the actual clinical scenario of BKPyV infection *in vivo*. Our results showed that MPyV DNA loads in the kidney peaked at approximately 12 days after primary infection, followed by persistent infection for more than 9 weeks. These results were consistent with the previous report (16).

BKPyVAN mainly occurred in kidney transplant recipients but rarely in other solid organ transplant recipients, although they all received potent IS (17). Thus, it seems possible that there are some renal allograft-specific factors involved in polyomavirus reactivation. One explanation is that the transplanted kidney underwent IRI and cellular regeneration processes. Accordingly, our results demonstrated that IRI alone can promote MPyV replication, which was in line with the clinical observations that prolonged ischemia time contributed to BKPyV infection (18). This also partially explained the increased trend of BKPyV infection in the era of deceased donor transplantation.

So how is IRI linked to polyomavirus replication? Our study showed that the most significant renal tubular epithelial cell damage appeared on day 3 after IRI, followed by cellular repair. This suggested that IRI and polyomavirus replication are closely linked, although the involved mechanisms are not yet clear. Actually, the limited genome size and coding capacity of polyomavirus determine that it has to utilize multiple host cell replication elements to complete the viral replication cycle (19, 20). BKPyV may “hijack” the mechanisms of kidney repair and regeneration to promote its own replication (21). We speculated that renal IRI initiated cell regeneration, thereby creating a more favorable microenvironment for polyomavirus replication (22). Besides, viral loads in the left kidney (clamped) were much higher than those in the right kidney (unclamped) in this study, suggesting that factors promoting MPyV replication were mainly localized in the kidney rather than in circulation.

In addition to IRI, IS has also been proved to be a risk factor for BKPyV infection. Berebbi et al. observed that the kidney of adult nude mice remained non-permissive for polyomavirus replication, indirectly indicating that suppressing host antiviral immunity may not be the only way by which IS promoted polyomavirus replication (23). Tacrolimus was reported to induce obvious renal tubular epithelial cell injury and cellular division (24). Hirsch et al. observed that tacrolimus could directly facilitate BKPyV replication, accompanied by an increase in host cellular DNA synthesis (25). These results, together with our findings, suggest that tacrolimus may provide a favorable microenvironment for polyomavirus replication by inducing cellular damage and subsequent DNA synthesis.

In clinical practice, tacrolimus is often not used alone but in combination with other immunosuppressive agents. The combination of tacrolimus and mycophenolate is currently the most commonly used immunosuppressive regimen in kidney transplant recipients. This regimen is most closely associated with polyomavirus infection after renal transplantation (26, 27). Therefore, the initial aim of this study was to use a combination of tacrolimus + mycophenolic acid to mimic the effect of a clinically realistic immunosuppressive regimen on promoting polyomavirus replication.

In addition to tacrolimus and mycophenolate, mTOR inhibitors were previously considered as a protective immunosuppressant in BKPyVAN. *In vitro* study shows that mTOR1 inhibitors can reduce the expression of the non-coding control region of wild-type and mutant polyomavirus (28). Molecular mechanism investigation revealed that mTOR1 inhibitor (sirolimus) impaired BKPyV replication by binding FK506-binding protein (FKBP12) and inhibiting mTOR-SP6-kinase activation (25). However, Alvarez et al. found that mTOR inhibitors increase polyomavirus large T-antigen expression and directly activate polyomavirus by inhibiting S-phase kinase-associated protein 2 E3 ligase (29). Therefore, the effect of mTOR inhibitors on polyomavirus is not well defined, and more studies are needed to investigate it in the future.

NF-κB signaling pathway has been documented to be involved in oxidative stress, cell differentiation, and tissue repair. In addition, NF-κB, as a transcription factor, also participates in regulating polyomavirus replication (30). A highly conserved sequence binding to NF-κB is present within the early leader region immediately upstream from the early gene initiation codon between nucleotides 9–22 and 25–34 of the BKPyV genome (31). Overexpression of the NF-κB p65 subunit enhanced the transcription level of the BKPyV early promoter (31). Both renal IRI and tacrolimus can induce NF-κB signaling activation (32, 33). Taken together, our data suggest that NF-κB signaling may be the common signaling pathway mediating IRI and IS to promote polyomavirus replication. Intragraft gene expression profiling of BKPyVAN (34) revealed significantly increased expression of complement factor D, which was a serine protease that cleaved C3b-bound factor B, resulting in the formation of the alternative pathway C3 convertase (35). Complement C3 can also activate NF-κB signaling (36), which was in agreement with our results that C3 was screened as a core gene.

Transcriptome analysis identified EGF, a multifunctional growth factor, as a potential core gene in the pathogenesis of BKPyVAN (37). The possible mechanism is that EGFR can activate the NF-κB signaling pathway (38).

Some studies have uncovered that FN1, a high-molecular-weight extracellular matrix protein, assisted in viral replication, metastasis, and entry, such as HBV (39). Besides, FN1 was reported to activate the NF-κB pathway and increase the nuclear localization of P65 (40, 41). Therefore, exploring the role of FN1 in regulating polyomavirus replication may bring unexpected findings.

Notably, although MPyV is similar to human BKPyV in terms of genetics, structure, prevalence, infectivity, and tropism, there are genomic differences between MPyV and BKPyV. For example, middle T antigen is encoded by MPyV but not BKPyV, while agnoprotein is expressed by BKPyV but not MPyV. These heterogeneities may lead to differences in pathogenicity and immunogenicity between the two viruses. It is for this reason that we compared the microarray datasets of human BKPyVAN and stable transplanted kidneys (**Figure 7**). The results showed that those core genes that may be involved in promoting MPyV replication were similarly increased in BKPyVAN.

The present study has some limitations. In this study, the core genes that promote MPyV replication were screened out by animal experiments and bioinformatics analysis. However, the extent to which these genes contribute to polyomavirus replication needs to be further investigated. Besides, viral genomes and structural proteins of MPyV and BKPyV are not 100% identical. These heterogeneities may lead to differences in pathogenicity and immunogenicity between the two viruses. The conclusions drawn from MPyV in mice cannot be directly extrapolated to BKPyV in humans.

In conclusion, this study investigating MPyV replication using a mouse model of MPyV infection can provide informative clues for further understanding the mechanisms of polyomavirus activation. Screening for specific inhibitors or blockers targeting these core genes will provide promising avenues for combating polyomavirus infection.

## Data Availability Statement

The data presented in the study are deposited in the Gene Expression Omnibus repository, accession number GSE192576.

## Ethics Statement

The animal study was reviewed and approved by the Ethics Committee of Guangdong Laboratory Animals Monitoring Institute (No. I-IACUC2019006).

## Author Contributions

All authors made an important contribution to the manuscript. GH and LZ designed the study. X-TC, YH, and GH interpreted the data and wrote the manuscript. S-CY, as a pathologist, evaluated the slides. YH, JW, and GL established the animal models and collected the samples. YZ and L-FH performed laboratory testing. JW and Y-XL were in charge of viral culture and identification. HZ and G-DZ performed the bioinformatics analysis. GH, LZ, and JQ revised and edited the whole manuscript. All of the authors have read and approved the manuscript.

## Funding

This work was supported by grants from the National Natural Science Foundation of China (81770749) and China Postdoctoral Science Foundation (2020M672982).

## Conflict of Interest

The authors declare that the research was conducted in the absence of any commercial or financial relationships that could be construed as a potential conflict of interest.

## Publisher’s Note

All claims expressed in this article are solely those of the authors and do not necessarily represent those of their affiliated organizations, or those of the publisher, the editors and the reviewers. Any product that may be evaluated in this article, or claim that may be made by its manufacturer, is not guaranteed or endorsed by the publisher.

## Abbreviations

BKPyV, BK polyomavirus; BKPyVAN, BK polyomavirus-associated nephropathy; EGFR, epidermal growth factor receptor; FN1, fibronectin 1; IRI, ischemia–reperfusion injury; IS, immunosuppressants; KEGG, Kyoto Encyclopedia of Genes and Genomes; MPyV, mouse polyomavirus; WGCNA, weighted gene correlation network analysis; PPI, protein–protein interaction; RNA-seq, RNA sequencing; TOM, topological overlap measure.

## References

[1] Mayberry CL Bond AC Wilczek MP Mehmood K Maginnis MS. Sending Mixed Signals: Polyomavirus Entry and Trafficking. Curr Opin Virol (2021) 47:95–105. doi: 10.1016/j.coviro.2021.02.004 33690104 PMC8068616

[2] Albrecht JA Dong Y Wang J Breeden C Farris AR Lukacher AE. Adaptive Immunity Rather Than Viral Cytopathology Mediates Polyomavirus-Associated Nephropathy in Mice. Am J Transplant (2012) 12:1419–28. doi: 10.1111/j.1600-6143.2012.04005.x PMC3365603 22420885

[3] Han LE Kemball CC Wang J Dong Y Stapler DJ Hamby KM. A Mouse Model for Polyomavirus-Associated Nephropathy of Kidney Transplants. Am J Transplant (2006) 6:913–22. doi: 10.1111/j.1600-6143.2006.01265.x 16611327

[4] Huang G Zeng G Huang Y Ramaswami B Randhawa P. Evaluation of the Gastrointestinal Tract as Potential Route of Primary Polyomavirus Infection in Mice. PloS One (2016) 11:e150786. doi: 10.1371/journal.pone.0150786 PMC4777556 26939117

[5] Chen S Zhou Y Chen Y Gu J. Fastp: An Ultra-Fast All-in-One FASTQ Preprocessor. Bioinformatics (2018) 34:i884–90. doi: 10.1093/bioinformatics/bty560 PMC6129281 30423086

[6] Patro R Duggal G Love MI Irizarry RA Kingsford C. Salmon Provides Fast and Bias-Aware Quantification of Transcript Expression. Nat Methods (2017) 14:417–9. doi: 10.1038/nmeth.4197 PMC5600148 28263959

[7] Langfelder P Horvath S. WGCNA: An R Package for Weighted Correlation Network Analysis. BMC Bioinf (2008) 9:559. doi: 10.1186/1471-2105-9-559 PMC2631488 19114008

[8] Kanehisa M Sato Y Kawashima M. KEGG Mapping Tools for Uncovering Hidden Features in Biological Data. Protein Sci (2022) 31:47–53. doi: 10.1002/pro.4172 34423492 PMC8740838

[9] Yu G Wang LG Han Y He QY. Clusterprofiler: An R Package for Comparing Biological Themes Among Gene Clusters. Omics (2012) 16:284–7. doi: 10.1089/omi.2011.0118 PMC3339379 22455463

[10] Chin CH Chen SH Wu HH Ho CW Ko MT Lin CY. Cytohubba: Identifying Hub Objects and Sub-Networks From Complex Interactome. BMC Syst Biol (2014) 8(Suppl 4):S11. doi: 10.1186/1752-0509-8-S4-S11 25521941 PMC4290687

[11] Sigdel TK Gao Y He J Wang A Nicora CD Fillmore TL. Mining the Human Urine Proteome for Monitoring Renal Transplant Injury. Kidney Int (2016) 89:1244–52. doi: 10.1016/j.kint.2015.12.049 PMC5221536 27165815

[12] Lubetzky M Bao Y O BP Marfo K Ajaimy M Aljanabi A. Genomics of BK Viremia in Kidney Transplant Recipients. Transplantation (2014) 97:451–6. doi: 10.1097/01.TP.0000437432.35227.3e 24310299

[13] Demey B Tinez C François C Helle F Choukroun G Duverlie G. Risk Factors for BK Virus Viremia and Nephropathy After Kidney Transplantation: A Systematic Review. J Clin Virol (2018) 109:6–12. doi: 10.1016/j.jcv.2018.10.002 30343190

[14] Saleh A El DKM Ezzat A Takou A Halawa A. Update on the Management of BK Virus Infection. Exp Clin Transplant (2020) 18:659–70. doi: 10.6002/ect.2019.0254 32552624

[15] Knowles WA. Discovery and Epidemiology of the Human Polyomaviruses BK Virus (BKV) and JC Virus (JCV). Adv Exp Med Biol (2006) 577:19–45. doi: 10.1007/0-387-32957-9_2 16626025

[16] Dubensky TW Villarreal LP. The Primary Site of Replication Alters the Eventual Site of Persistent Infection by Polyomavirus in Mice. J Virol (1984) 50:541–6. doi: 10.1128/jvi.50.2.541-546.1984 PMC255666 6323753

[17] Hirsch HH Randhawa P. BK Polyomavirus in Solid Organ Transplantation. Am J Transplant (2013) 13(Suppl 4):179–88. doi: 10.1111/ajt.12110 23465010

[18] Bressollette-Bodin C Coste-Burel M Hourmant M Sebille V Andre-Garnier E Imbert-Marcille BM. A Prospective Longitudinal Study of BK Virus Infection in 104 Renal Transplant Recipients. Am J Transplant (2005) 5:1926–33. doi: 10.1111/j.1600-6143.2005.00934.x 15996241

[19] Ambalathingal GR Francis RS Smyth MJ Smith C Khanna R. BK Polyomavirus: Clinical Aspects, Immune Regulation, and Emerging Therapies. Clin Microbiol Rev (2017) 30:503–28. doi: 10.1128/CMR.00074-16 PMC5355639 28298471

[20] Yang JF You J. Regulation of Polyomavirus Transcription by Viral and Cellular Factors. Viruses (2020) 12:1072. doi: 10.3390/v12101072 PMC7601649 32987952

[21] Mayberry CL Maginnis MS. Taking the Scenic Route: Polyomaviruses Utilize Multiple Pathways to Reach the Same Destination. Viruses (2020) 12:1168. doi: 10.3390/v12101168 PMC7602598 33076363

[22] Rochford R Moreno JP Peake ML Villarreal LP. Enhancer Dependence of Polyomavirus Persistence in Mouse Kidneys. J Virol (1992) 66:3287–97. doi: 10.1128/jvi.66.6.3287-3297.1992 PMC241106 1316448

[23] Demengeot J Jacquemier J Torrente M Blangy D Berebbi M. Pattern of Polyomavirus Replication From Infection Until Tumor Formation in the Organs of Athymic Nu/Nu Mice. J Virol (1990) 64:5633–9. doi: 10.1128/jvi.64.11.5633-5639.1990 PMC248619 2170689

[24] Ferjani H El AA Bouraoui A Achour A Abid S Bacha H. Protective Effect of Mycophenolate Mofetil Against Nephrotoxicity and Hepatotoxicity Induced by Tacrolimus in Wistar Rats. J Physiol Biochem (2016) 72:133–44. doi: 10.1007/s13105-015-0451-7 26746208

[25] Hirsch HH Yakhontova K Lu M Manzetti J. BK Polyomavirus Replication in Renal Tubular Epithelial Cells Is Inhibited by Sirolimus, But Activated by Tacrolimus Through a Pathway Involving FKBP-12. Am J Transplant (2016) 16:821–32. doi: 10.1111/ajt.13541 PMC5064607 26639422

[26] Hirsch HH Vincenti F Friman S Tuncer M Citterio F Wiecek A. Polyomavirus BK Replication in *De Novo* Kidney Transplant Patients Receiving Tacrolimus or Cyclosporine: A Prospective, Randomized, Multicenter Study. Am J Transplant (2013) 13:136–45. doi: 10.1111/j.1600-6143.2012.04320.x PMC3563214 23137180

[27] Brennan DC Agha I Bohl DL Schnitzler MA Hardinger KL Lockwood M. Incidence of BK With Tacrolimus Versus Cyclosporine and Impact of Preemptive Immunosuppression Reduction. Am J Transplant (2005) 5:582–94. doi: 10.1111/j.1600-6143.2005.00742.x 15707414

[28] Korth J Anastasiou OE Verheyen J Dickow J Sertznig H Frericks N. Impact of Immune Suppressive Agents on the BK-Polyomavirus Non Coding Control Region. Antiviral Res (2018) 159:68–76. doi: 10.1016/j.antiviral.2018.09.013 30268912

[29] Alvarez OJ Kwun HJ Artusi S Chang Y Moore PS. Sirolimus and Other Mechanistic Target of Rapamycin Inhibitors Directly Activate Latent Pathogenic Human Polyomavirus Replication. J Infect Dis (2021) 224:1160–9. doi: 10.1093/infdis/jiaa071 PMC8514189 32060513

[30] White MK Bellizzi A Ibba G Pietropaolo V Palamara AT Wollebo HS. The DNA Damage Response Promotes Polyomavirus JC Infection by Nucleus to Cytoplasm NF- kappaB Activation. Virol J (2017) 14:31. doi: 10.1186/s12985-017-0707-7 28202068 PMC5312431

[31] Gorrill TS Khalili K. Cooperative Interaction of P65 and C/EBPbeta Modulates Transcription of BKV Early Promoter. Virology (2005) 335:1–9. doi: 10.1016/j.virol.2005.02.006 15823601

[32] Reid S Scholey J. Recent Approaches to Targeting Canonical Nfκb Signalling in the Early Inflammatory Response to Renal IRI. J Am Soc Nephrol (2021) 32(9):2117–24. doi: 10.1681/ASN.2021010069 PMC8729839 34108233

[33] González-Guerrero C Ocaña-Salceda C Berzal S Carrasco S Fernández-Fernández B Cannata-Ortiz P. Calcineurin Inhibitors Recruit Protein Kinases JAK2 and JNK, TLR Signaling and the UPR to Activate NF-κb-Mediated Inflammatory Responses in Kidney Tubular Cells. Toxicol Appl Pharmacol (2013) 272:825–41. doi: 10.1016/j.taap.2013.08.011 23958496

[34] Sigdel TK Bestard O Salomonis N Hsieh SC Torras J Naesens M. Intragraft Antiviral-Specific Gene Expression as a Distinctive Transcriptional Signature for Studies in Polyomavirus-Associated Nephropathy. Transplantation (2016) 100:2062–70. doi: 10.1097/TP.0000000000001214 PMC5235336 27140517

[35] Ricklin D Hajishengallis G Yang K Lambris JD. Complement: A Key System for Immune Surveillance and Homeostasis. Nat Immunol (2010) 11:785–97. doi: 10.1038/ni.1923 PMC2924908 20720586

[36] Mastellos DC Deangelis RA Lambris JD. Complement-Triggered Pathways Orchestrate Regenerative Responses Throughout Phylogenesis. Semin Immunol (2013) 25:29–38. doi: 10.1016/j.smim.2013.04.002 23684626 PMC3920450

[37] Jia L Fu W Jia R Wu L Li X Jia Q. Identification of Potential Key Protein Interaction Networks of BK Virus Nephropathy in Patients Receiving Kidney Transplantation. Sci Rep (2018) 8:5017. doi: 10.1038/s41598-018-23492-2 29567951 PMC5864740

[38] Zhang L Zhang J Chen Z Wang L Wu X Ou M. Epidermal Growth Factor (EGF) Triggers the Malignancy of Hemangioma Cells *via* Activation of NF-κb Signals. BioMed Pharmacother (2016) 82:133–40. doi: 10.1016/j.biopha.2016.05.002 27470348

[39] Lee WY Bachtiar M Choo C Lee CG. Comprehensive Review of Hepatitis B Virus-Associated Hepatocellular Carcinoma Research Through Text Mining and Big Data Analytics. Biol Rev Camb Philos Soc (2019) 94:353–67. doi: 10.1111/brv.12457 30105774

[40] Wang J Deng L Huang J Cai R Zhu X Liu F. High Expression of Fibronectin 1 Suppresses Apoptosis Through the NF-κb Pathway and is Associated With Migration in Nasopharyngeal Carcinoma. Am J Transl Res (2017) 9:4502–11. PMC5666059 29118912

[41] Zhang H Chen X Xue P Ma X Li J Zhang J. FN1 Promotes Chondrocyte Differentiation and Collagen Production *via* TGF-β/PI3K/Akt Pathway in Mice With Femoral Fracture. Gene (2021) 769:145253. doi: 10.1016/j.gene.2020.145253 33098939

