# Supplementary material for: Ischemia–Reperfusion Injury and Immunosuppressants Promote Polyomavirus Replication Through Common Molecular Mechanisms
Source: Front Immunol. 2022 Feb 25;13:835584. doi: 10.3389/fimmu.2022.835584 (PMC8914341; doi:10.3389/fimmu.2022.835584)
Supplement: Supplementary file 1 [file DataSheet_1.docx]

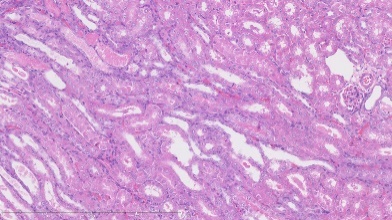


B


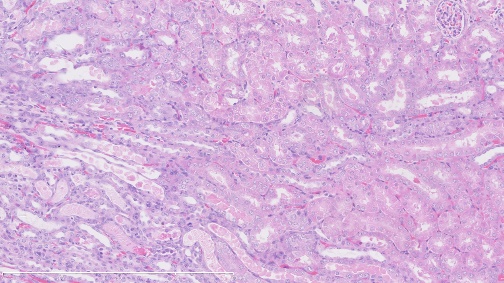


A


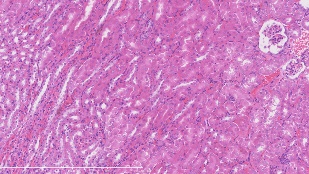


D


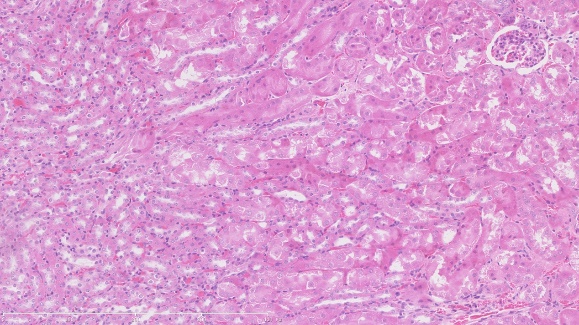


C

Supplemental Figure 1. Dynamic pathological manifestations after IRI in the left kidney of C57BL/6 mice (Hematoxylin and Eosin staining). (A) On day 3, acute tubular injury was found through the cortical and medullary areas. The brushing border of proximal tubules was shed off. The lumen enlarged and granular casts were identified in the distant and collective tubules of the medulla. (B) On day 7, area of acute tubular injury was diminished. The proximal tubules were swollen with some epithelium showing shed-off brush borders and scattered calcification. The area between cortex and medulla still showed foci of acute tubular injury with fewer granular casts than in Day 3. (C) On day 14, only mild swelling was found in the tubular epithelium. (D) On day 21, the renal tissue was recovered from IRI and no evidence of acute tubular injury was observed.

Abbreviations: ischemia-reperfusion injury (IRI)
