# Supplementary material for: Ischemia–Reperfusion Injury and Immunosuppressants Promote Polyomavirus Replication Through Common Molecular Mechanisms
Source: Front Immunol. 2022 Feb 25;13:835584. doi: 10.3389/fimmu.2022.835584 (PMC8914341; doi:10.3389/fimmu.2022.835584)
Supplement: Supplementary file 2 [file Table_1.docx]

**Supplemental Table 1.** Kinetics of viral loads in left kidney of C57BL/6 mice at multiple time points among groups after primary mouse polyomavirus infection

| Groups | MPyV DNA (log_10_ copies/mg) | | | | |
| --- | --- | --- | --- | --- | --- |
|  | 3 days | 7 days | 10 days | 14 days | 21 days |
| Control group | 4.420 ± 0.414 | 4.230 ± 0.263 | 4.430 ± 0.313 | 4.085 ± 0.287 | 4.010 ± 0.147 |
| IRI group | 6.118 ± 0.934 | 4.880 ± 0.704 | 5.205 ± 0.341 | 4.530 ± 1.269 | 4.025 ± 0.679 |
| IS group | 5.643 ± 0.602 | 4.730 ± 1.069 | 5.815 ± 0.191 | 5.428 ± 0.723 | 5.368 ± 0.164 |

Abbreviations: mouse polyomavirus (MPyV), ischemia-reperfusion injury (IRI), immunosuppressant (IS)
