# Supplementary material for: Ischemia–Reperfusion Injury and Immunosuppressants Promote Polyomavirus Replication Through Common Molecular Mechanisms
Source: Front Immunol. 2022 Feb 25;13:835584. doi: 10.3389/fimmu.2022.835584 (PMC8914341; doi:10.3389/fimmu.2022.835584)
Supplement: Supplementary file 3 [file Table_2.docx]

Supplemental Table 2. Correspondence between core genes and enrichment pathways

| Genes | KEGG pathways |
| --- | --- |
| C3 | NF-kappa B signaling pathway |
| FN1 | ECM-receptor interaction |
| EGFR | Calcium signaling pathway, Protein digestion and absorption, and Neuroactive ligand-receptor interaction |

Abbreviations: fibronectin 1 (FN1), epidermal growth factor receptor (EGFR), extracellular matrix (ECM)
